# Permanent Draft Genome Sequence of *Frankia* sp. Strain AvcI1, a Nitrogen-Fixing Actinobacterium Isolated from the Root Nodules of *Alnus viridis* subsp. *crispa* Grown in Canada

**DOI:** 10.1128/genomeA.01511-15

**Published:** 2015-12-31

**Authors:** Erik Swanson, Rediet Oshone, Stephen Simpson, Krystalynne Morris, Feseha Abebe-Akele, W. Kelley Thomas, Louis S. Tisa

**Affiliations:** University of New Hampshire, Durham, New Hampshire, USA

## Abstract

*Frankia* strain AvcI1, isolated from root nodules of *Alnus viridis* subsp. *crispa*, is a member of *Frankia* lineage Ia, which is able to reinfect plants of the *Betulaceae* and *Myricaceae* families. Here, we report a 7.7-Mbp draft genome sequence with a G+C content of 72.41% and 6,470 candidate protein-encoding genes.

## GENOME ANNOUNCEMENT

*Frankia* spp. are filamentous nitrogen-fixing actinobacteria that are found as free-living microbes in the soil and in symbiotic association with dicotyledonous plants, termed actinorhizal plants (1–3). Actinorhizal plants are found worldwide in a broad range of ecological and environmental conditions (4), and this symbiosis allows them to colonize harsh environmental terrains. Although the genus *Frankia* has not yet been described to the species level, it has become an area of greater interest. Based on phylogenetic analysis, *Frankia* strains have been classified into four main lineages (5–8). Members of lineage 1 are found infective on host plants of the *Betulaceae*, *Myricaceae*, and *Casuarinaceae* families, while lineage 2 represents strains that are infective on *Rosaceae*, *Coriariaceae*, *Datiscaceae*, and the genus *Ceanothus* (*Rhamnaceae*). Members of lineage 3 are the most promiscuous and are infective on *Eleagnaceae*, *Rhamnaceae*, *Myricaceae*, *Gynmnostoma*, and occasionally *Alnus*. The fourth *Frankia* lineage consists of the “atypical” strains that are unable to reinfect actinorhizal host plants or form ineffective root nodule structures that are unable to fix nitrogen. Genomes for representatives from each cluster have been sequenced (9–21). Analysis of *Frankia* genomes has revealed new potential in respect to metabolic diversity, natural product biosynthesis, and stress tolerance, which may help aid the cosmopolitan nature of the actinorhizal symbiosis.

Cluster I contains two subclusters: one subcluster (cluster Ia) represents *Frankia* strains with the ability to infect a wider range of host plants, including members of the *Betulaceae* and *Myricaceae* families, and the other subcluster (cluster Ib) contains strains limited to *Casuarina* and *Allocasuarina* host plants. As another member of cluster Ia, *Frankia* sp. strain AvcI1 was chosen for sequencing to provide more information on this lineage and its interaction with actinorhizal plants. *Frankia* sp. strain AvcI1 was isolated from root nodules of *Alnus viridis* subsp. *crispa* collected from Atikokan, Ontario, Canada (22, 23).

The draft genome of *Frankia* sp. strain AvcI1 was generated at the Hubbard Genome Center (University of New Hampshire, Durham, NH, USA) using Illumina technology (24) techniques. A standard Illumina shotgun library was constructed and sequenced using the Illumina HiSeq2000 platform, which generated 27,505,182 reads (260-bp insert size) totaling 4,125.8 Mbp. The Illumina sequence data were assembled using CLC Genomics Workbench version 8.0.1 and AllPaths-LG version r41043 (25). The final draft assembly contained 77 contigs with an *N*_50_ of 107.5 kb. The total size of the genome is 7.7 Mbp, and the final assembly is based on 4,125.1 Mb of Illumina draft data and provided an average 399× coverage of the genome.

The high-quality draft genome of *Frankia* sp. strain AvcI1 was resolved to 7,741,902 bp with a G+C content of 72.41%. The assembled *Frankia* sp. strain AvcI1 genome was annotated via the Integrated Microbial Genomes (IMG) platform developed by the Joint Genome Institute, Walnut Creek, CA, USA (26, 27) and resulted in 6,470 candidate protein-encoding genes, 46 tRNA genes, and 2 rRNA regions.

### Nucleotide sequence accession numbers.

This whole-genome shotgun project has been deposited at DDBJ/EMBL/GenBank under the accession number LJFZ00000000. The version described in this paper is the first version, LJFZ01000000.
